# Anti-inflammatory activity of 3-cinnamoyltribuloside and its metabolomic analysis *in LPS-activated RAW 264.7 cells*

**DOI:** 10.1186/s12906-020-03115-y

**Published:** 2020-11-02

**Authors:** Zhennan Wang, Ying Guan, Rui Yang, Junjian Li, Junsong Wang, Ai-Qun Jia

**Affiliations:** 1 School of Environmental and Biological Engineering, Nanjing University of Science and Technology, Nanjing, 210094 China; 2 School of Life and Pharmaceutical Sciences, Key Laboratory of Tropical Biological Resources of Ministry Education, Hainan University, Haikou, 570228 China; 3 Inspection and Pattern Evaluation Department, Suzhou Institute of Metrology, Suzhou, 215000 China

**Keywords:** 3-cinnamoyltribuloside, Anti-inflammatory, Cholinergic anti-inflammatory pathway, Metabolomics, RAW 264.7

## Abstract

**Background:**

Inflammation is a response to tissue injuries, which is indispensable and important for human health, but excessive inflammation can potentially cause damage to the host organisms. *Camellia nitidissima* Chi, one traditional medicinal and edible plant in China, was reported to exhibit anti-inflammation capability. Hence, this study was conducted to isolate the bioactive compounds from the flowers of *C. nitidissima* Chi and evaluate their anti-inflammatory activity.

**Methods:**

The phytochemicals from the flowers of *C. nitidissima* Chi were isolated and purified by silica gel, Sephadex LH-20 gel, C18 reversed silica gel, semi-preparative HPLC, and identified by the spectrum technologies. The anti-inflammatory activity of isolated compounds was evaluated using cultured macrophage RAW 264.7 cells. Whereafter the potential metabolic mechanism of the anti-inflammatory activity of the bioactive compound was investigated by a ^1^H-NMR based metabolomics approach. The metabolites in ^1^H-NMR spectra were identified by querying the Human Metabolome Database and Madison Metabolomics Consortium Database online. And the multivariate statistical analysis was performed to evaluate the variability of metabolites among samples and between sample classes.

**Results:**

The compound isolated from the flowers of *C. nitidissima* Chi was identified as 3-cinnamoyltribuloside (3-CT). 3-CT could inhibit the NO production and the mRNA expression of iNOS involved in lipopolysaccharide (LPS)-activated RAW 264.7 cells. Moreover, 3-CT could inhibit the expression of a series of inflammatory cytokines, including TNF-α, IL-1β, and IL-6, both at the mRNA level and protein level. The ^1^H-NMR based metabolomics approach was applied to investigate the potential metabolic mechanism of the anti-inflammatory activity of 3-CT. Thirty-five metabolites were identified and assigned. Orthogonal signal correction partial least-squares discriminant analysis (OSC-PLS-DA) of the ^1^H-NMR data showed 3-CT could balance the significant changes in many endogenous metabolites (e.g., choline, glucose, phenylalanine) induced by LPS in RAW 264.7 cells, which related to cholinergic anti-inflammatory pathway, oxidative stress, energy metabolism, and amino acids metabolism.

**Conclusion:**

3-CT, isolated from the flowers of *C. nitidissima* Chi, had potent anti-inflammatory activity in LPS-activated RAW 264.7 cells. Furthermore, our results indicated that 3-CT had effects on the cholinergic anti-inflammatory pathway, oxidative stress, energy metabolism, and amino acids metabolism in LPS-activated RAW 264.7 cells.

**Supplementary Information:**

The online version contains supplementary material available at 10.1186/s12906-020-03115-y.

## Background

Inflammation is part of the complex and essential biological process responding to tissue mechanical injuries, biological stimuli, or due to aberrant autoimmune response [1, 2]. Although inflammation is indispensable and important for homeostasis and human health, excessive inflammation can potentially cause damage for the host organisms and induce many diseases, such as cancer, rheumatoid arthritis, diabetes, septic shock, and cardiovascular diseases due to the toxic effects of several inflammatory factors [1, 3]. Macrophages, which have two distinguishable phenotypes, classical/pro-inflammatory (M1) and alternative/anti-inflammatory (M2), play an important role in the immune responses to infection in the inflammatory process [4, 5]. Lipopolysaccharide (LPS), one of the outer cell membrane components of gram-negative bacteria, is a famous endotoxin which can activate macrophages to produce many kinds of inflammatory cytokines such as tumor necrosis factor-α (TNF-α), interleukin-1β (IL-1β), and interleukin-6 (IL-6) etc. [6, 7]. Besides, LPS can also activate macrophages to produce inflammatory mediators, like nitric oxide (NO) [1, 7]. Therefore, the inhibition of LPS activated macrophages to release cytokines and mediators is always used to develop and evaluate new anti-inflammatory agents.

In recent years, drug development has often relied on the use of natural and synthetic drugs for the prevention or treatment of many diseases [8]. Compared with synthetic drugs, natural anti-inflammatory ingredients attract more and more attention due to their weak side effects [1, 9]. Currently, many natural ingredients have been put into clinical use as anti-inflammatory drugs, such as triptolide, resveratrol, silymarin [10, 11]. The medicinal and edible plants in China are rich in anti-inflammatory components, especially flavonoids that have the activity of multiple anti-inflammatory [12, 13]. Flavonoids are usually via interfering with nuclear factor kappa B (NFκB) and mitogen-activated protein kinase (MAPK) to control inflammatory signaling cascades and inhibit the formation of inflammatory cytokines, such as TNF-α, IL-1β, and IL-6 [14–16].

*Camellia nitidissima* Chi, distributed in Southern China and Northern Vietnam, is one traditional medicinal and edible plant in China. Our previous researches showed that the *C. nitidissima* Chi flowers had high total phenolic content, and played a potential role in human health, especially *C. nitidissima* Chi flowers could inhibit the formation of advanced glycation end-products [17], showed the anti-quorum sensing [18] and antioxidant [19] activities. Besides, *C. nitidissima* Chi was also reported to exhibit anti-inflammation capability [20]. However, it is not known if the flowers of *C. nitidissima* Chi, the rarest part of the species, have anti-inflammatory activity, and if so, what anti-inflammatory compound(s) contribute(s) to such an activity.

3-cinnamoyltribuloside (3-CT), a rich source of cinnamolyglycoflavonoid, possess a broad range of biological activities, including antioxidant, antimycobacterial, and cytotoxicity [21–23]. In this study, 3-CT isolated from the flowers of *C. nitidissima* Chi firstly, which showed potent anti-inflammatory activity in LPS-activated RAW 264.7 cells under bioassays. Furthermore, we evaluated the anti-inflammatory metabolic mechanism via ^1^H-NMR-based metabolomics after 3-CT treatment.

## Methods

### Chemicals and reagents

Lipopolysaccharides, (3–4,5-dimethylthiazol-2-yl)-2,5-diphenyl tetrazolium bromide (MTT), dimethyl sulfoxide (DMSO), and sodium 3-(trimethylsilyl)-propionic acid (TSP) were purchased from Sigma (St. Louis, MO, USA). Dulbecco’s modified eagle medium (DMEM), fetal bovine serum (FBS), and penicillin/ streptomycin were obtained from GIBCO (Grand Island, NY, USA). Trypsin was purchased from Solarbio science and technology co., Ltd. (Beijing, China). The nitric oxide assay kit was obtained from Nanjing Jiancheng Bioengineering Institute (Nanjing, China). Mouse tumor necrosis factor-alpha, interleukin 1β, and interleukin 6 ELISA kit were purchased from Multi Sciences biotechnology Co., Lid. (Hangzhou, China). Trizol Reagent was purchased from Invitrogen (Carlsbad, CA, USA). Acetonitrile (analytical grade) was purchased from Merck (Darmstadt, Germany). Deuterium oxide (D_2_O, 99.9%) was purchased from Sea Sky Bio Technology Co., Ltd. (Beijing, China). Other chemical reagents were purchased from Nanjing Shuangling Chemical Reagent Co., Ltd. (Nanjing, China).

### The isolation and purification of 3-CT from ethyl acetate fraction

*C. nitidissima* Chi flowers were collected in July 2016 from a planting base in Fangchenggang, China. Then the ethyl acetate fraction of extracts from *C. nitidissima* Chi flowers was obtained following our previous study [19].

Next, the ethyl acetate fraction was repeatedly subjected to different column chromatographies, such as silica gel (200–300 meshes, Qingdao Marine Chemical Inc.), Sephadex LH-20 (GE Healthcare Bio-Sciences AB, Uppsala, Sweden), and C_18_ reversed silica gel (YMC, Japan), to yield 3-CT (83 mg). And ^1^H- and ^13^C-NMR spectra were recorded on a Bruker AV-500 (Bruker Inc., Germany). ESI-MS was conducted on Thermo TSQ Quantum LC/MS spectrometers (USA).

### Cell culture

A murine macrophage cell line RAW 264.7 was a kind gift from Dr. Yuan Xu from Nanjing University of Chinese Medicine (Nanjing, China). Cells were grown in high-glucose DMEM supplemented with 10% FBS, and 1% antibiotics (penicillin and streptomycin) in a humidified incubator flushed continuously at 37 °C with 5% CO_2_. The culture medium was refreshed every day.

### MTT assay for RAW 264.7cell cytotoxicity

MTT assay was conducted as published methods [24], with some modifications. Firstly, 3-CT was dissolved in DMSO, and then diluted into different concentrations with the culture medium. The final DMSO concentration in all assays did not exceed 0.1% (v/v). Cells were seeded in a 96 well plate at a density of 5 × 10^3^ cells/well. After 24 h, the cells were treated with various concentrations of 3-CT for 24 h, and the final concentrations of 3-CT were 0.625, 1.25, 2.5, 5, 10, 20, and 40 μM. DMSO (final concentration of 0.1% (v/v)) served as a negative control, and cells without treatment served as the blank control. After 24 h treatment, cells were incubated with 5 mg/mL MTT solution for 4 h at 37 °C and 5% CO_2_. Then the medium was removed, and 150 μL DMSO was added to dissolve the precipitation. Absorbance at 490 nm was determined using an ELISA reader (TECAN Infinite 200 Pro, Switzerland).

### Nitric oxide assay

Production of NO by LPS-activated RAW 264.7 cells was measured by using the NO assay kit (Nanjing Jiancheng Bioengineering Institute). RAW 264.7 cells were incubated at 5 × 10^3^ cells/well in 96 well-plates for 24 h at 37 °C and 5% CO_2_. And then the cells were treated with various concentrations of 3-CT in the presence of LPS (1 μg/mL). The final concentrations of 3-CT were 0.3125, 0.625, 1.25, 2.5, 5, and 10 μM. The cells without treatment served as the blank control. The cells with LPS + DMSO (final concentration of 0.1% (v/v)) in the absence of 3-CT served as the negative control (LPS group). After 24 h treatment, NO was analyzed following the manufacturer’s protocols.

### Quantitative real-time PCR analysis

The quantitative real-time PCR procedures were performed according to previous studies [4], with some modifications. RAW 264.7 cells were seeded into 6 well-plates for 24 h at 37 °C and 5% CO_2_. And then the cells were treated with various concentrations (0.625, 1.25, 2.5, 5, and 10 μM) of 3-CT in the presence of LPS (1 μg/mL). The cells without treatment served as the blank control. The cells with LPS + DMSO (final concentration of 0.1% (v/v)) in the absence of 3-CT served as the negative control (LPS group). After 24 h treatment, the total RNA was extracted with Trizol Reagent according to the manufacturer’s recommended procedure. The RNA was then reverse transcribed into complementary DNA (cDNA) using commercial reverse transcript enzyme (KeyGene, Nanjing, China) according to the manufacturer’s instructions. The real-time RT-PCR was performed in a 20 μL volume using an SYBR Green PCR Core Reagent Kit (Vazyme, China) according to the manufacturer’s instructions by Applied Biosystems 7300 RT-PCR System (USA). The relative expression of each gene was calculated by the 2^−ΔΔCT^ method normalized to *Gapdh*. Forward and reverse primers used for this study are listed in Table 1.

**Table 1:** The sequences of primers used for quantitative real-time PCR

Gene	Sense (5′-3′)	Anti-sense (5′-3′)
IL-1β	TGGAAAAGCGGTTTGTCTTC	TACCAGTTGGGGAACTCTGC
IL-6	GAGGATACCACTCCCAACAGACC	AAGTGCATCATCGTTGTTCATACA
iNOS	CAGGAGGAGAGAGATCCGATTTA	GCATTAGCATGGAAGCAAAGA
TNF-α	CATCTTCTCAAAATTCGAGTGAC	TGGGAGTAGACAAGGTACAACCC
GAPDH	GGCCTTCCGTGTTCCTAC	TGTCATCATATCTGGCAGGTT

### Measurements of TNF-α, IL-1β, IL-6 by ELISA

TNF-α, IL-1β, and IL-6 produced by LPS-activated RAW 264.7 cells were determined by using mouse TNF-α, IL-1β, and IL-6 ELISA kit, respectively. RAW 264.7 cells were incubated at 5 × 10^3^ cells/well in 96 well-plates for 24 h at 37 °C and 5% CO_2_. And then the cells were treated with various concentrations (0.3125, 0.625, 1.25, 2.5, 5, and 10 μM) of 3-CT in the presence of LPS (1 μg/mL). The cells without treatment served as the blank control. The cells with LPS + DMSO (final concentration of 0.1% (v/v)) in the absence of 3-CT served as the negative control (LPS group). After 24 h treatment, TNF-α, IL-1β, and IL-6 were analyzed following the manufacturer’s protocols.

### Metabolite extraction of LPS-activated RAW 264.7 cells

Metabolites of LPS-activated RAW 264.7 cells were extracted according to the previous study [25], with some modifications. RAW 264.7 cells were incubated 100-mm-diameter culture dish for 24 h at 37 °C and 5% CO_2_, one dish as one sample for ^1^H-NMR analysis. And then three 3-CT treated groups were conducted at the concentrations of 2.5, 5, and 10 μM corresponding to the low, medium, and high dosages in the presence of LPS (1 μg/mL). The cells without treatment served as the blank control. The cells with LPS + DMSO (final concentration of 0.1% (v/v)) in the absence of 3-CT served as the negative control (LPS group).

After 24 h treatment, the cells were washed with PBS three times. Then, the cells were scraped off in ice-cold solution of acetonitrile/water (1:1, v/v, 2 ml per dish). Subsequently, from the obtained cell suspension, the intracellular metabolites were extracted through ultrasonication for 20 min. Then, the mixtures were centrifuged at 12,000 rpm and at 4 °C for 15 min. Next, the supernatant was frozen at − 80 °C overnight and then lyophilized in a freeze drier.

### ^1^H-NMR measurements of extracts from LPS-activated RAW 264.7 cells

The lyophilized extracts were rehydrated in 600 μL of 99.8% D_2_O phosphate buffer (pH 7.4), which contained 0.05% (w/v) TSP. The mixture solutions were vortexed and centrifuged to eliminate the precipitates. Then the afforded transparent supernatants were shifted into NMR tubes for ^1^H-NMR analysis.

The ^1^H-NMR spectra of samples were acquired on a Bruker AVANCE III 500 MHz NMR spectrometer, using the Carr-Purcell-Meiboom-Gill sequence [90(τ-180-τ)n-acquisition] with a total spin-echo delay (2 nτ) of 40 ms to suppress the signals of proteins. The following parameters: controlling temperature of 298 K, the spectral width of 10,000 Hz, data points of 32 K. The D_2_O provided a lock signal and the TSP was used as the chemical shift reference (^1^H, 0.00 ppm). The free induction decays were weighted using an exponential window function corresponding to a line-broadening of 0.5 Hz before a Fourier transformation.

### ^1^H-NMR data processing and analysis of metabolites from LPS-activated RAW 264.7 cells

Before analysis, the ^1^H-NMR spectra were phase- and baseline- corrected manually and referenced to the chemical shift of TSP (*δ* 0.0 ppm) using Topspin 3.0 software (version 3.0, Bruker GmbH, Karlsruhe, Germany). Subsequently, all the spectra were exported to ASCII files using MestReNova (Version 8.0.1, Mestrelab Research SL) and then read into R software (https://www.r-project.org/) for multivariate analysis. Each spectrum was divided into 0.005 ppm wide segments between 0.2 and 10 ppm. The segments between 4.3 and 5.8 ppm were cut off to exclude the residual signal of water. All spectra were then normalized and mean-centered prior to multivariate statistical analysis.

The metabolites in ^1^H-NMR spectra were identified by querying the Human Metabolome Database (HMDB, http://www.hmdb.ca) and Madison Metabolomics Consortium Database (MMCD, http://mmcd.nmrfam.wisc.edu) online, aided by the Chenomx NMR suite 7.5 (Chenomx Inc., Edmonton, Canada) and further confirmed by statistical total correlation spectroscopy (STOCSY) [26].

Multivariate statistical analysis, including principal component analysis (PCA) and supervised orthogonal signal correction partial least-squares discriminant analysis (OSC-PLS-DA), were performed to evaluate variability among samples and between sample classes. Initially, unsupervised PCA was applied to reduce the dimensionality of imported ^1^H-NMR data by transforming to new latent variables, namely, principal components, to summarize the features of the data. Moreover, the supervised OSC-PLS-DA technique can eliminate irrelevant effects and maximize the distinction of intergroup differences. The OSC was applied prior to PLS-DA to filter out unrelated variables that do not contribute to class discrimination to minimize the influence of unrelated signals.

Repeated two-fold cross-validation and permutation test (2000 times) was conducted to evaluate the validation of the OSC-PLS-DA model. The parameters for the explained variation, R^2^, and the predictive capability, Q^2^, indicated the excellence of the constructed models [26]. Color-coded loading plots were constructed to demonstrate which variables are responsible for group separation. The fold-change values of metabolites were highlighted by using color-code in colored tables as well as their *p*-values adjusted by the Benjamini-Hochberg method [27].

Univariate analysis was performed using the parametric Student’s *t-*test and nonparametric Mann-Whitney test [28] to estimate significant differences in crucial metabolites. The fold-change values of the identified metabolites and their associated *p-*values between groups were calculated. And then the obtained *p-*values were corrected using the Benjamini-Hochberg method to control the false discovery rate in multiple comparisons.

### Statistical analysis

All the data were presented as means ± standard deviation or averages and all experiments were conducted independently in triplicate. Analysis of variance (ANOVA) with Dunnett test was performed through SPSS version 17.0 (SPSS Inc., Chicago, IL, USA) software. Statistical significance was determined at *p* < 0.05.

## Results

### Elucidation of 3-CT

**Fig. 1:**
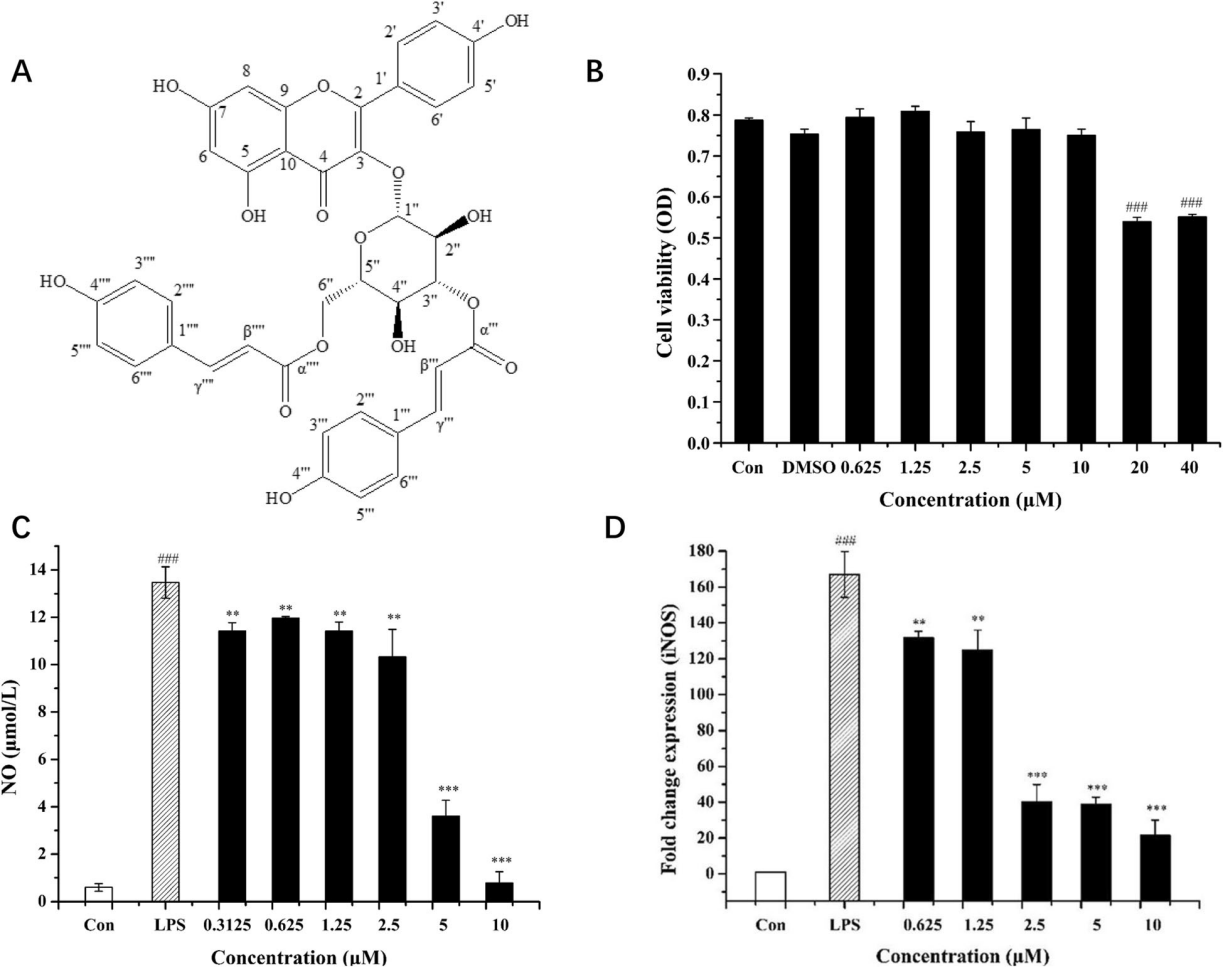
Effect of 3-CT on NO production and INOS mRNA Expression in LPS-activated RAW 264.7 cells. **a** Structure of 3-CT. **b** Effects of 3-CT on the RAW 264.7 cells viability were determined by MTT assay. **c** Effects of 3-CT on NO production in LPS-activated RAW 264.7 cells were measured by NO assay kit. **d** Effects of 3-CT on mRNA expressions of iNOS were determined by quantitative real-time PCR analysis. The values are expressed as the means ± SD. ### *p* < 0.001, compared with the control group. *p < 0.05, **p < 0.01, ***p < 0.001, compared with the LPS group, *n* = 3

The spectral data of 3-CT were as follows: ESI-MS, *m/z* 739.09 [M-H]^−^. ^1^H-NMR (500 MHz, CD_3_OD) *δ*:7.96 (2H, d, *J* = 8.8 Hz, H-2′, 6′), 7.73 (1H, d, *J* = 15.9 Hz, H-γ”’), 7.49 (2H, d, *J* = 8.6 Hz, H-2″’, 6″’), 7.41 (1H, d, *J* = 15.9 Hz, H-γ”“), 7.30 (2H, d, *J* = 8.6 Hz, H-2”“, 6”“), 6.86 (2H, d, *J* = 8.6 Hz, H-3”’, 5″’), 6.81 (2H, d, *J* = 8.6 Hz, H-3″“, 5”“), 6.80 (2H, d, *J* = 8.2 Hz, H-3’, 5’), 6.49 (1H, *J* = 15.9 Hz, H-β”’), 6.24 (1H, s, H-8), 6.09 (1H, *J* = 15.9 Hz, H-β”“), 6.04 (1H, *J* = 1.7 Hz, H-6), 5.68 (1H, *J* = 8.0 Hz, H-1”), 5.12 (1H, t, *J* = 9.2 Hz, H-3″), 4.39 (1H, *J* = 11.5 Hz, H_a_-6″), 4.24 (1H, dd, *J* = 11.5, 6.6 Hz, H_b_-6″), 3.74 (1H, m, H-4″), 3.64 (1H, m, H-5″), 3.47 (1H, m, H-2″). ^13^C-NMR (125 MHz, CD_3_OD) *δ*: 178.8 (C-4), 168.9 (C-α”’), 168.8 (C-α”“), 165.2 (C-7), 162.5 (C-5), 161.0 (C-4”’), 160.9 (C-4”“), 160.8 (C-4’), 158.8 (C-2), 158.0 (C-9), 147.1 (C-γ”’), 146.4 (C-γ”“), 134.4 (C-3), 132.1 (C-2’, 6’), 131.2 (C-2”“, 6”“), 131.1 (C-2”’, 6″’), 127.2 (C-1″’), 126.9 (C-1”“), 122.8 (C-1’), 116.7 (C-3”“, 5”“), 116.6 (C-3”’, 5″’), 116.0 (C-3′, 5′), 115.2 (C-β”’), 114.6 (C-β”“), 105.7 (C-10), 100.4 (C-1”), 99.7 (C-6), 94.7 (C-8), 75.9 (C-3″), 75.5 (C-5″), 75.6 (C-4″), 71.8 (C-2″), 64.4 (C-6″). All spectral data were in agreement with a report [21]. So it was identified as 3-cinnamoyltribuloside, which was isolated from *C. nitidissima* Chi flowers for the first time (Fig. 1a).

### Effects of 3-CT on cell viability in RAW 264.7 cells

The potential effects of 3-CT on cell viability in RAW 264.7 cells were analyzed by the MTT assay after incubating cells for 24 h without LPS. As shown in Fig. 1b, when the concentrations of 3-CT were 0.625–10 μΜ, RAW 264.7 cells viability were not decreased within 24 h, and at the concentrations of 20 μΜ and 40 μΜ, 3-CT could markedly (*p* < 0.001) suppress the RAW 264.7 cells viability. So 3-CT had no cytotoxicity on RAW 264.7 cells at the concentrations of 0.625–10 μΜ.

### Effects of 3-CT on NO production in LPS-activated RAW 264.7 cells

In this study, NO production was measured in the culture medium to determine the anti-inflammatory activity of 3-CT. LPS-activated RAW 264.7 cells were treated with 3-CT at the concentrations of 0.3125–10 μΜ. As shown in Fig. 1c, 3-CT showed inhibitory effects on the NO production at all tested concentrations, especially at 10 μΜ, the inhibition of NO production was 98.57%. And even at the concentration 0.3125 μΜ, the inhibition of NO production was 15.87%, which showed that the NO production was significantly (*p* < 0.01) inhibited compared with the LPS-treated group (Fig. 1c).

Furthermore, we investigated the inhibitory activity of 3-CT on the LPS-activated iNOS mRNA expression in RAW 264.7 cells. As shown in Fig. 1d, compared with the control, the expression of iNOS mRNA was significantly (*p* < 0.001) increased when cells were stimulated by LPS. In contrast, treatment of 3-CT at the concentrations of 0.625–10 μΜ all inhibited the mRNA expression of iNOS, with the strongest inhibitory effect at the concentration of 10 μΜ.

### Effects of 3-CT on the production of inflammatory cytokines

**Fig. 2:**
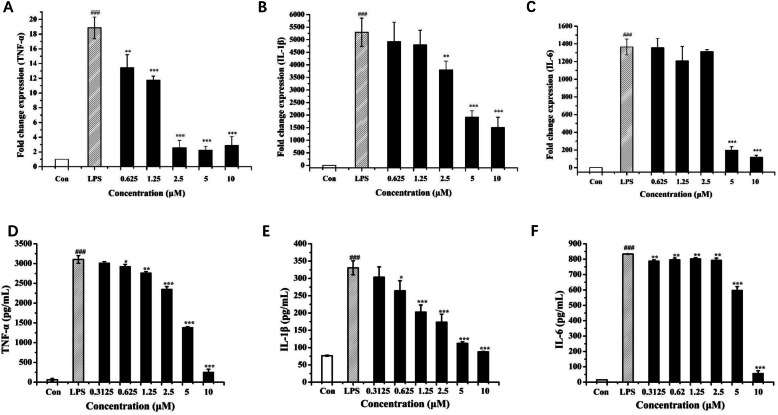
Effects of 3-CT on the production of TNF-α, IL-1β and IL-6 in LPS-activated RAW 264.7 cells. Effects of 3-CT on mRNA expressions of TNF-α (**a**), IL-1β (**b**) and IL-6 (**c**) were determined by quantitative real-time PCR analysis. ELISA was performed to examine protein levels of TNF-α (**d**), IL-1β (**e**) and IL-6 (**F**) cytokines. The values are expressed as the means ± SD. ### *p* < 0.001, compared with the control. **p < 0.01, ***p < 0.001, compared with the LPS group, *n* = 3

Firstly, we investigated whether 3-CT could affect the mRNA expression of TNF-α, IL-1β, and IL-6, and the results were shown in Fig. 2a-c. At the concentrations of 0.625–10 μΜ, 3-CT could inhibit the mRNA expression of TNF-α (Fig. 2a) compared with the LPS group. However, only at higher concentrations (2.5 μΜ, 5 μΜ, and 10 μΜ), did 3-CT suppress the mRNA expression of IL-1β (Fig. 2b) and display little effect on IL-1β expression at lower concentrations (0.625 μΜ and 1.25 μΜ). Similarly, at the concentrations of 0.625 μΜ, 1.25 μΜ, and 2.5 μΜ, 3-CT had no significant effects on the mRNA expression of IL-6, whereas, at the concentrations of 5 μΜ and 10 μΜ, 3-CT could significantly (*p* < 0.001) inhibit the mRNA expression of IL-6 (Fig. 2c).

Secondly, we measured the release of TNF-α, IL-1β, and IL-6 in the culture medium by ELISA Kit. As shown in Fig. 2d-f, at the concentrations of 0.625–10 μΜ, 3-CT had remarkable inhibitory effects on TNF-α, IL-1β, and IL-6, respectively. Especially at 10 μΜ 3-CT, the inhibition of the release of all of the three inflammatory cytokines was strongest in LPS-activated RAW 264.7 cells, which was similar to the results of their mRNA expressions.

### Metabolite assignment and multivariate data analysis

**Fig. 3:**
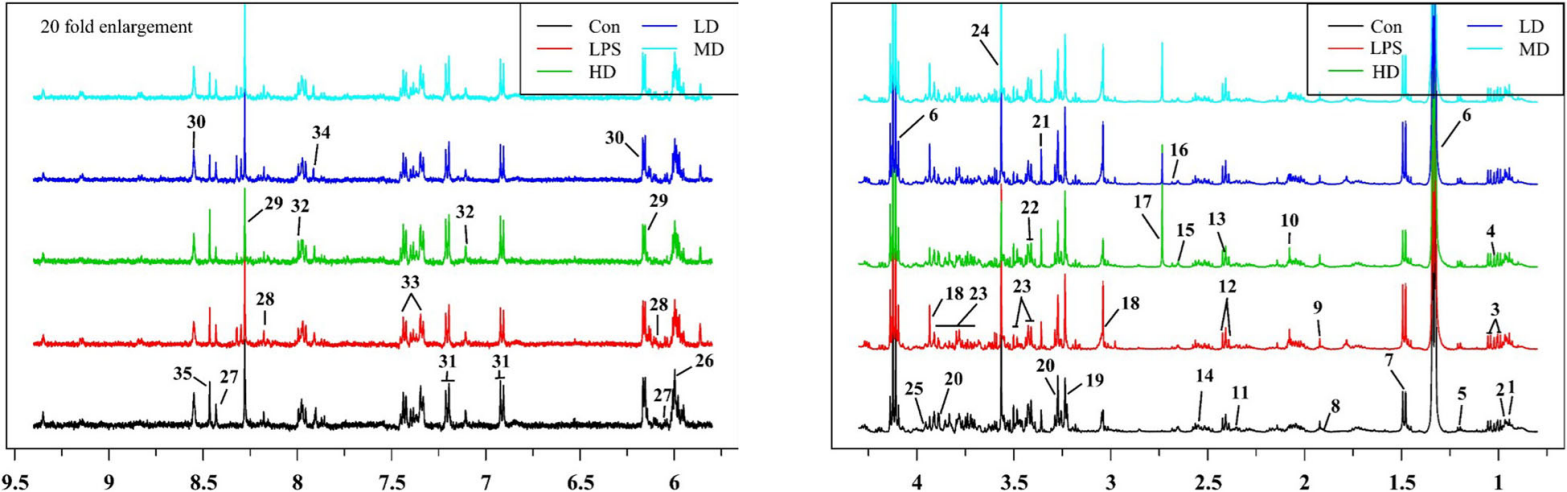
Typical 500 MHz ^1^H-NMR spectra of RAW 264.7 cells with the metabolites labeled. Metabolites:1, 2-Aminobutyrate; 2, Leucine; 3, Valine; 4, Isoleucine; 5, Ethanol; 6, Lactate; 7, Alanine; 8, Lysine; 9, Acetate; 10, Homoserine; 11, Glutamate; 12, Pyroglutamat; 13, Succinate; 14, Glutathione; 15, 5, 6-Dihydrouracil; 16, Sarcosine; 17, Dimethylamine; 18, Creatine; 19, Choline; 20, Betaine; 21, Methanol; 22, Taurine; 23, Glucose; 24, Glycine; 25, Serine; 26, Cytosine; 27, NAD^+^; 28, NADP^+^; 29, AMP; 30, ATP; 31, Tyramine; 32, Histamine; 33, Phenylalanine; 34, Histidine; 35, Formate

**Fig. 4:**
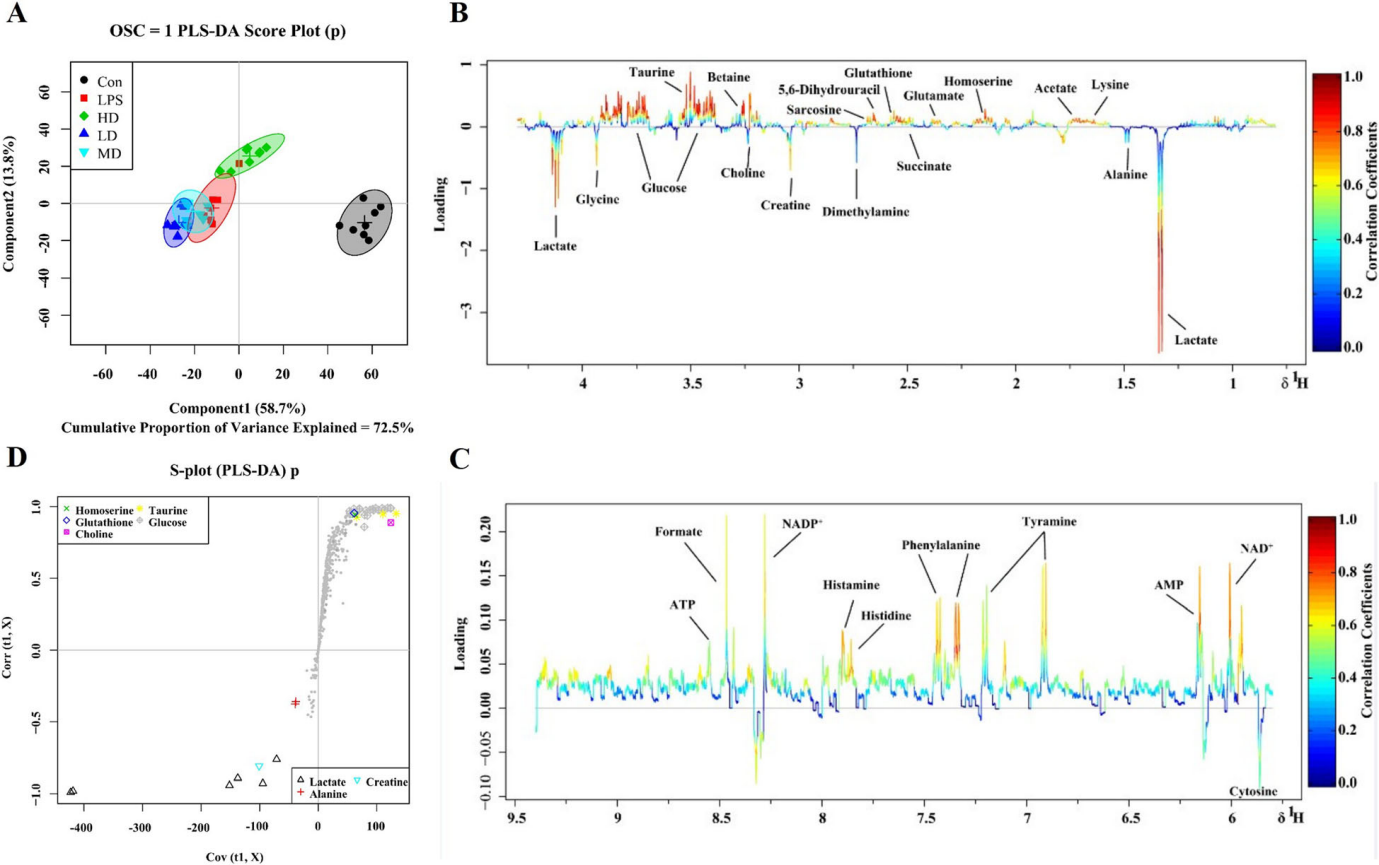
OSC-PLS-DA analysis of NMR data from the five groups of RAW 264.7 cells. **a** Score plot., in which Component 1 (58.7%) and component 2 (13.8%) explained 72.5% of total variance in the five groups of RAW 264.7 sample extracts, indicated the discriminations among the five groups. **b**, **c** Color-coded loadings plots. Color bar was applied, with red and blue representing metabolites that significantly or indistinctively contributed to the separation of groups, respectively. Peaks in positive and negative status reveal decreased and increased metabolites, respectively, relative to the score plot in the 3-CT-treated group. **d** S-plot indicated the metabolites responsible for the significantly discrimination among these groups

Representative 500 MHz CPMG ^1^H-NMR spectra obtained from Con, LPS, HD, MD, and LD groups were shown in Fig. 3. From these spectrums, 35 metabolites identified and their detailed information is shown in Table 2, including their fold change value and *p-*values. The STOCSY technique was used to identify the correlation between spectral resonances of interest to assist the assignment of metabolites, such as lactate, dimethylamine, tyramine, and formate (Fig. S1). PCA score plot showed a clear separation between LPS-alone and 3-CT treated groups and doses groups overlapped with each other (Fig. S2). The supervised OSC-PLS-DA was then performed to reveal the significant changed metabolic among the five groups. The OSC-PLS-DA score plot (Fig. 4a) displays a clear separation between the control group and the LPS group, and similarly LD, MD groups showed significant discrimination with the LPS group. The metabolites responsible for the significant discrimination among these groups were demonstrated in S-plot (Fig. 4d) and the color-coded loading plots (Fig. 4b, c).

**Table 2:**
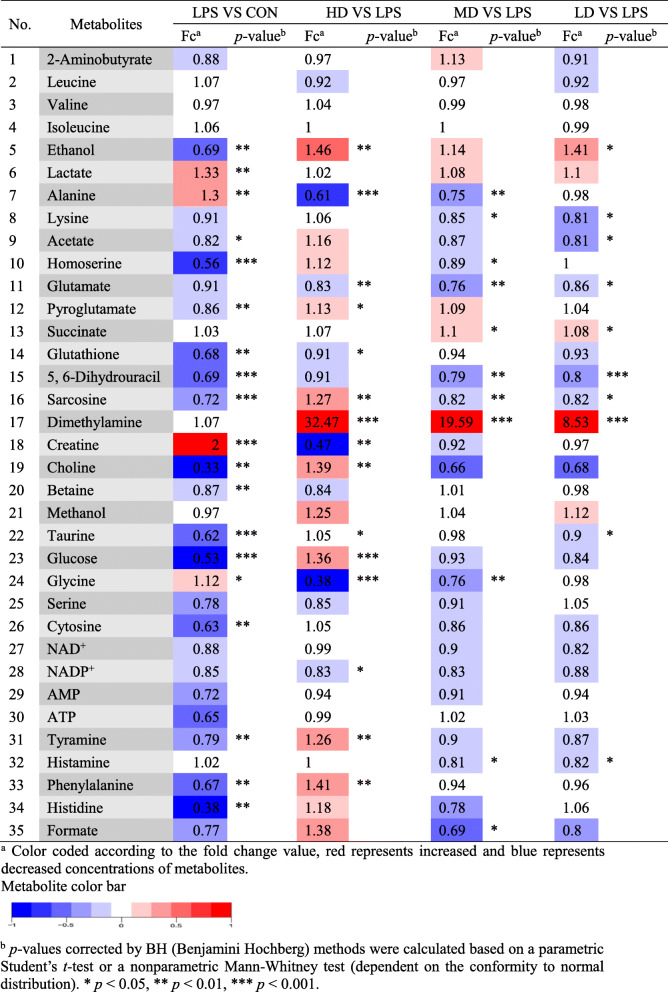
Metabolites in RAW 264.7 cells identified by ^1^H-NMR and their fold changes among groups and the associated *p*-values

The major metabolites partly representing disease status were selected to evaluate the anti-inflammatory effects of 3-CT. As shown in Table 2, the metabolites were quantified and illustrated, the LPS group had higher levels of alanine, creatine, glycine than the control group, but their levels were obviously reduced after 3 -CT treatment. Moreover, relative to the control group, the levels of ethanol, pyroglutamate, sarcosine, choline, taurine, glucose, tyramine, phenylalanine were significantly declined in the LPS group. Noteworthy, 3-CT treatment markedly increased these metabolites in RAW 264.7 cells. However, other metabolites did not show a significant recovery trend.

## Discussion

**Fig. 5:**
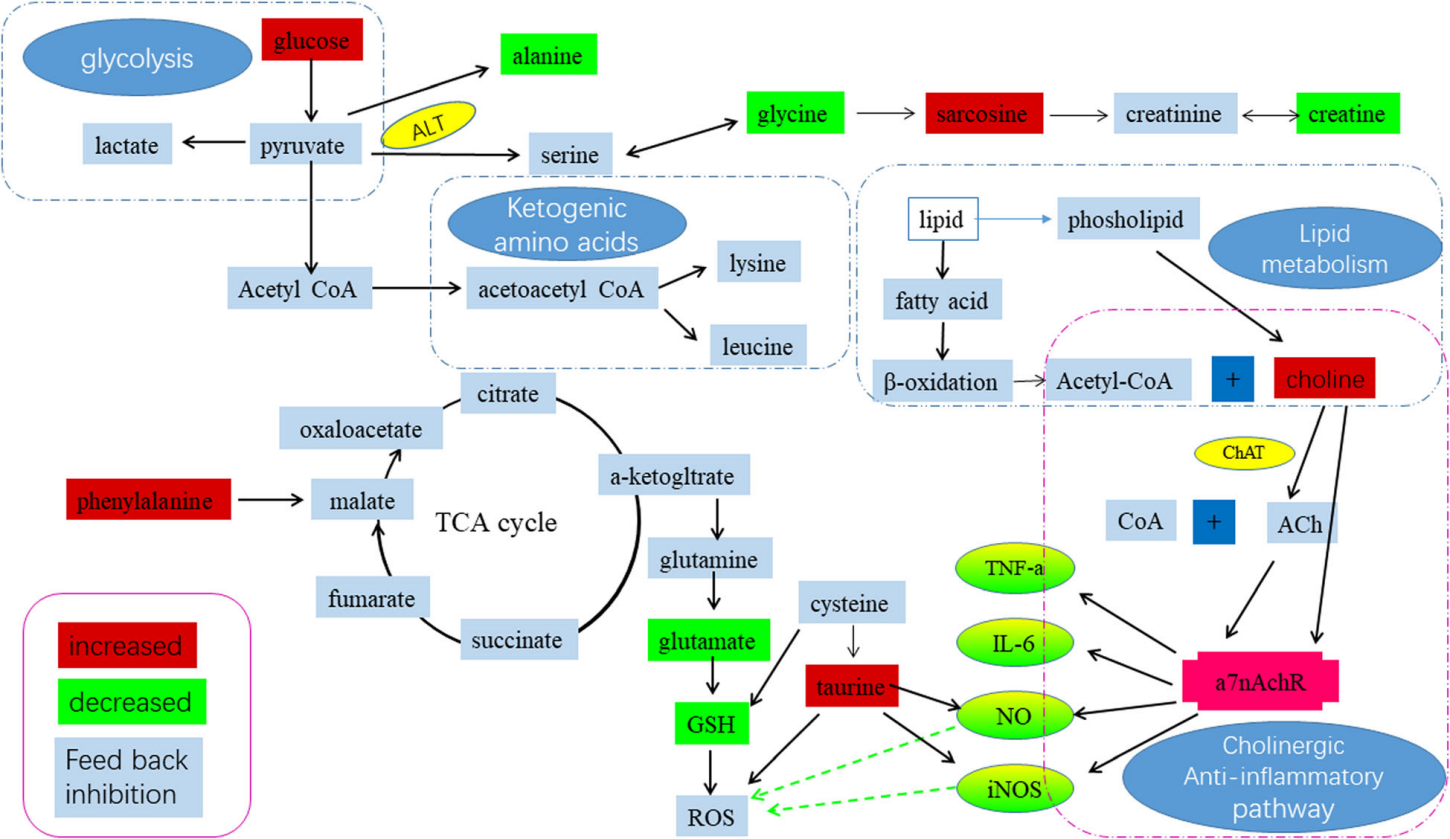
Schematic diagram of the main metabolic pathways and signaling pathways in response to inflammation induced by LPS and the treatment effects of 3-CT on inflammation in RAW.264.7 cells, showing the interrelationship of the identified metabolic pathways

In this study, 3-CT was isolated from *C. nitidissima* Chi flowers firstly, and its potent anti-inflammatory activity was evaluated in LPS-activated RAW 264.7 cells. 3-CT exhibited significant anti-inflammatory activity by inhibiting the production of inflammatory factors and mediators in LPS-activated RAW 264.7 cells. In addition, the ^1^H-NMR-based metabolomics approach combined with multivariate statistical analysis was applied to examine the metabolic responses to 3-CT treatment in LPS-activated RAW 264.7 cells. The significantly changed levels of metabolites induced by LPS in RAW 264.7 cells, such as the increase of levels of alanine, creatine, glycine and the decrease of the levels of ethanol, pyroglutamate, sarcosine, choline, taurine, glucose, tyramine, phenylalanine, were reversed after 3- CT treatment, suggesting a good balance effect of 3-CT against LPS induced metabolic disorders in RAW 264.7 cells. Furthermore, the potential metabolic mechanism of the anti-inflammatory activity of 3-CT was preliminarily clarified base on significant changes in many endogenous metabolites, which are related to the cholinergic anti-inflammatory pathway, oxidative stress, energy metabolism, and amino acids metabolism in LPS-activated RAW 264.7 cells. Based on these findings, perturbed metabolic pathways were proposed (Fig. 5).

### Anti-inflammation

NO plays a central role in diverse inflammatory and excessive NO could trigger deleterious consequences such as chronic inflammation, carcinogenesis, and sepsis [7, 29]. And previous studies had shown that NO is generated from L-arginine by the enzymatic action of three highly homologous nitric oxide synthase (NOS): endothelial (eNOS), neuronal (nNOS), and inducible (iNOS). And each isoform had separate functions. Activated macrophages transcriptionally express iNOS, which is responsible for the prolonged and excessive production of NO. So some compounds might suppress the synthesis of iNOS protein or inhibit iNOS activity to decrease the NO production [30, 31]. In this study, we found that NO production and iNOS mRNA expression was inhibited in LPS-activated RAW264.7 cells after 3-CT treatment. So the results indicated that 3-CT could inhibit the NO production by suppression of the (over-)expression of iNOS to show excellent anti-inflammatory activity.

Macrophages, stimulated by LPS, could release a series of inflammatory cytokines, including TNF-α, IL-1β, and IL-6, all of which were involved in a variety of immunological reactions [32]. So the inhibitory effects of compounds on inflammatory cytokines in LPS-activated RAW 264.7 cells were always used to evaluate the activity of anti-inflammatory agents [1, 33]. Our results showed that, after 3-CT (10 μΜ) treatment, the expression of a series of inflammatory cytokines, including TNF-α, IL-1β, and IL-6, both at mRNA level and protein level, were significantly decreased. Based on these findings, we infered that 3-CT had inhibitory effects on the production of inflammatory cytokines (TNF-α, IL-1β, and IL-6), by interfering the mRNA expression and the release of inflammatory cytokines.

### Cholinergic anti-inflammatory pathway

The cholinergic anti-inflammatory pathway could be stimulated by electrical [34] or medicine methods, like nicotine, the drug AR-R17779 [35, 36] to regulate the inflammatory response and reduce the inflammatory damage. It could suppress systemic levels of TNF and other pro-inflammatory cytokines. And choline was an important component of the cholinergic anti-inflammatory pathway, which might reflect the anti-inflammatory effects of some medicines via a change in the level of choline [37–39]. Ma et al. found that saikosaponins which had anti-inflammatory activity could mitigate the imbalance of serum level of choline in inflammation mice model induced by formalin [40]. Similar results were also found in our study. As shown in Table 2, in the LPS group, choline markedly decreased compared with the control group, whereas in the HD group, choline significantly increased compared with the LPS group. In addition, it was reported that choline treatment stimulates the synthesis and release of acetylcholine (Ach) [41] and Ach could reduce the expression of TNF-α, IL-1β, IL-6 in the cholinergic anti-inflammatory pathway [42]. In this study, 3-CT enhances the choline level in LPS-activated RAW 264.7 cells, which facilitates the synthesis of Ach. Therefore, the result indicated that 3-CT shows the anti-inflammatory activity, possibly, attributed to its regulation of the cholinergic anti-inflammatory pathway.

### Oxidative stress

Oxidative stress could be considered as an imbalance between the production of reactive oxygen species (ROS) and the ability of the organism’s natural protective mechanisms to cope with these reactive compounds and prevent adverse effects, in which antioxidative enzymes played a key regulatory role to keep a certain stats of homeostasis [28]. Glutathione (GSH) redox system was a primary defense mechanism, which was important for maintaining the redox balance [43]. Compared with the LPS group, glutathione was significantly decreasing in the HD group, and glutamate remarkably decreased in HD, MD, and LD groups. The changes with treatment with 3-CT indicated that glutamate and glutathione were excessively consumed to balance the oxidative stress after being activated by LPS in RAW 264.7 cells. And this result revealed that 3-CT could not restore equilibrium to the GSH redox system and even slightly exacerbate the imbalance.

In inflammation, the excessive formation of NO could react with ROS to generate peroxynitrite that is implicated in oxidative damage [44], so the inhibitory effects of 3-CT on NO production could decrease the oxidative damage in LPS-activated RAW 264.7 cells.

Cell membranes are vulnerable to oxidative damage caused by unsaturated fatty acids [45]. Whereas, choline and phosphocholine are essential for maintaining the structural integrity of cell membranes. Compared with the control group, choline remarkably decreased in the LPS group, and in the HD group, choline significantly increased compared with the LPS group. The results indicated that when activated by LPS, choline was consumed to defend against the oxidative damage in RAW 264.7 cells, and by the treatment with 3-CT, the consumption of choline was decreased which might result from the reduction of the oxidative stress in RAW 264.7 cells. As an antioxidant, taurine showed protective effects against oxidative stress [46, 47]. Similar to choline, compared with the control group, taurine remarkably decreased in the LPS group, and in the HD group, taurine significantly increased compared with the LPS group. So the changes of taurine also showed that 3-CT could decrease the oxidative damage in LPS-activated RAW 264.7 cells. And similar results had also been found that flavonoids from *Glycyrrhiza* exhibited anti-inflammatory effects on treating arthritis in mice while attenuated the decrease of taurine level in urine [48].

Taken together, 3-CT had an effect on the levels of metabolites (glutathione, NO, choline, taurine) related to oxidative stress in RAW 264.7 cells. And it indicated the anti-inflammatory activity of 3-CT might be related to these effects.

### Energy metabolism

In inflammation, more energy must be generated for the accelerated metabolism due to the acute inflammatory insult [39, 49]. In this study, in the LPS group, glucose remarkably decreased compared with the control group, so it showed that large amounts of glucose were consumed to produce energy after RAW 264.7 cells were activated by LPS. But by the treatment with 3-CT, the level of glucose significantly increased in the HD group compared with the LPS group. This result is consistent with those of previous studies. Yao et al. detected a slight increase in plasm glucose levels in mice after volatile oil treatment relative to the model group [50]. Therefore it could speculate that 3-CT showed the anti-inflammatory effect in LPS-activated RAW 264.7 cells, which resulted in the decrease of energy demand, then the consumption of glucose remarkably decreased. However, it was notable that the level of ATP did not increase, even decreased (no statistic significance), when glucose was consumed to produce energy after RAW 264.7 cells were activated by LPS. Based on this result, it could speculate that the energy produced from glucose could not satisfy the increasing energy demand.

When the tricarboxylic acid cycle was inhibited, other sources were called for the enhancement energy supply, such as anoxic respiration, glycolysis, where pyruvate could generate energy by being metabolized to lactate and alanine [39, 51]. Compared with the control group, lactate and alanine both significantly increased in the LPS group, and in HD and MD groups, alanine remarkably decreased compared with the LPS group. However, anaerobic respiration is not as effective as aerobic respiration in energy production, which could not produce enough energy to meet the energy requirements of cells stimulated by LPS. This might be the reason why the level of ATP did not rise after 3-CT treatment and it also indicated 3-CT did not restore aerobic respiration to produce enough energy in LPS-activated RAW 264.7 cells.

So we could conclude that the energy metabolism of RAW 264.7 cells was disturbed after the induction by LPS. By the treatment with 3-CT, the energy metabolism in anaerobic respiration became balanced might due to its anti-inflammatory activity, which could reduce the energy demand of LPS-activated RAW 264.7 cells.

### Amino acids metabolism

Phenylalanine was an essential amino acid and involved in the synthesis of many important functional proteins [52, 53]. Compared with the control group, the level of phenylalanine remarkably decreased in the LPS group, which reflected the effects of LPS on muscle protein metabolism in RAW 264.7 cells. After treated by 3-CT, the level of phenylalanine significantly increased in the HD group compared with the LPS group. These results showed that 3-CT had the ability to balance phenylalanine metabolism in LPS-activated RAW 264.7 cells. Besides, as shown in Table 2, the levels of alanine, lysine, homoserine, sarcosine, glycine, and histidine all showed significant changes following the treatment with 3-CT. Amino acids play important roles both as basic substrates and as regulators in many metabolic pathways [54]. So it further concluded that 3-CT might show the anti-inflammatory activity through affecting amino acids metabolism in LPS-activated RAW 264.7 cells.

## Conclusions

In this study, 3-CT was isolated from *C. nitidissima* Chi flowers for the first time exhibiting potent anti-inflammatory activity in LPS-activated RAW 264.7 cells. To our best knowledge, it was the first time to report the anti-inflammatory activity of 3-CT. 3-CT could inhibit the NO production and mRNA expression of iNOS involved in LPS-activated RAW 264.7 cells. Furthermore, the results of mRNA expression and ELISA assay indicated that 3-CT also had the ability to inhibit the production of inflammatory cytokines, including TNF-α, IL-1β, and IL-6 in LPS-activated RAW 264.7 cells. The potential metabolic mechanism of the anti-inflammatory activity of 3-CT was explored by using ^1^H-NMR based metabolomics approach and 35 metabolites were identified via the CPMG ^1^H-NMR spectra. The significant change metabolites were further confirmed by using multivariate statistical analysis. Our results illustrated that 3-CT had effects on cholinergic anti-inflammatory pathway, oxidative stress, energy metabolism, and amino acids metabolism in LPS-activated RAW 264.7 cells.

## Supplementary information

**Additional file 1: Figure S1.** Example of two-dimensional statistical total correlation spectroscopy (STOCSY) analysis of ^1^H-NMR spectrum RAW 264.7 extracts to facilitate the identification of metabolites. (A) 2D STOCSY subplots from 5.8 to 9.4 ppm for the assignments of formate and tyramine; (B) 2D STOCSY subplots from 0.8 to 4.3 ppm for the assignments of dimethylamine and lactate.

**Additional file 2: Figure S2.** PCA scores plot for Con, LPS, HD, MD and LD groups.

**Additional file 3: Figure S3.** ^1^H-NMR spectra of 3-CT.

**Additional file 4: Figure S4.** ^13^C-NMR spectra of 3-CT.

**Additional file 5: Table S1.** The characteristic chemical shifts of metabolites in ^1^H-NMR spectra.

## Data Availability

The datasets used and/or analyzed during the current study available from the corresponding author on reasonable request.

## Abbreviations

3-CT: 3-cinnamoyltribuloside
LPS: Lipopolysaccharide
TNF-α: Tumor necrosis factor-α
IL-1β: Interleukin-1β
IL-6: Interleukin-6
NO: Nitric oxide
MTT: (3–4,5-dimethylthiazol-2-yl)-2,5-diphenyl tetrazolium bromide
DMSO: Dimethyl sulfoxide
TSP: Sodium 3-(trimethylsilyl)-propionic acid
DMEM: Dulbecco’s modified eagle medium
FBS: Foetal bovine serum
PCA: Principal component analysis
OSC-PLS-DA: Supervised orthogonal signal correction partial least-squares discriminant analysis
STOCSY: Statistical total correlation spectroscopy
