# Supplementary figures and images for: Anti-inflammatory activity of 3-cinnamoyltribuloside and its metabolomic analysis in LPS-activated RAW 264.7 cells
Source: BMC Complement Med Ther. 2020 Nov 2;20:329. doi: 10.1186/s12906-020-03115-y (PMC7607671; doi:10.1186/s12906-020-03115-y)

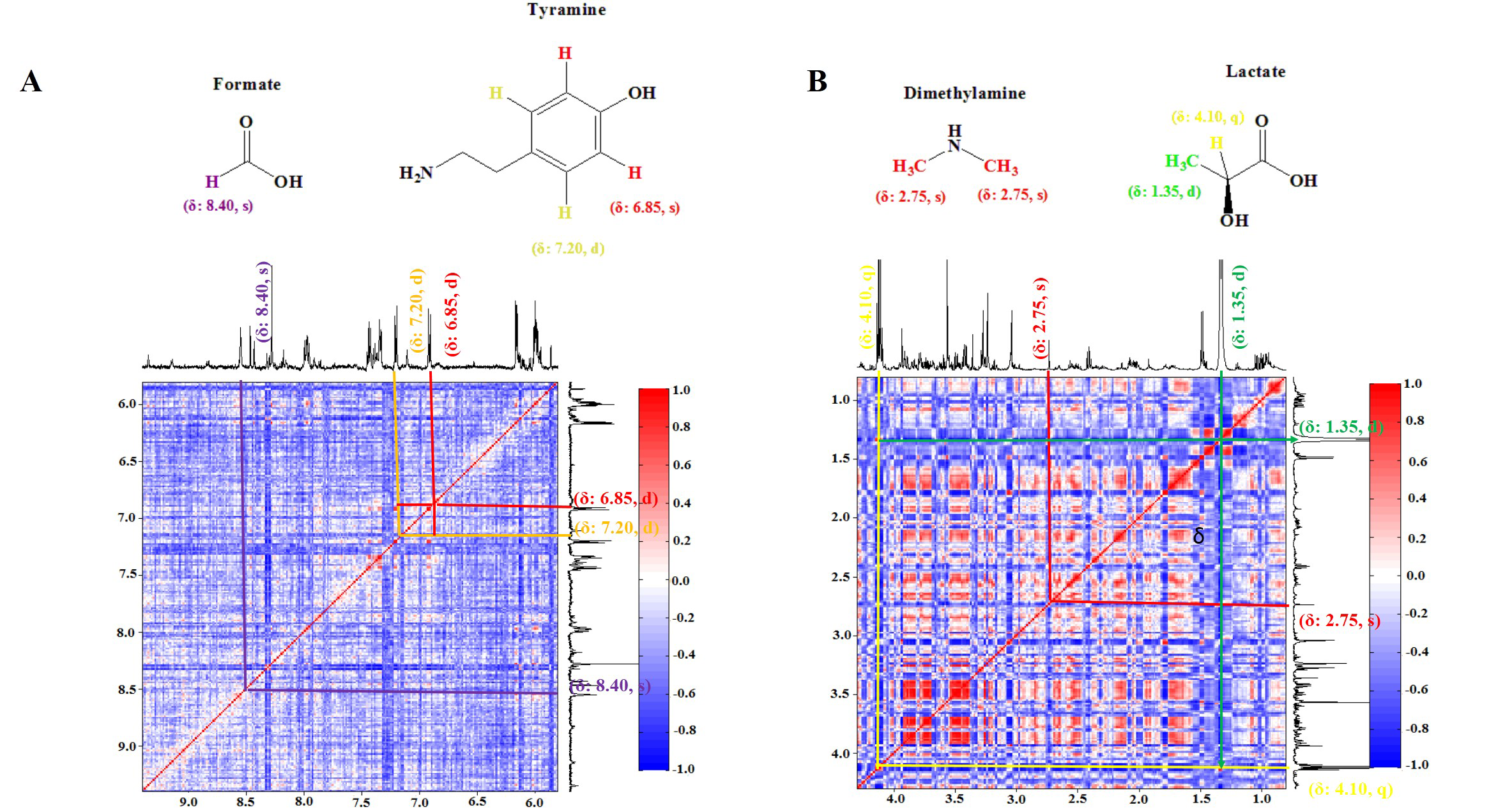

Supplement: Supplementary file 1 — Additional file 1: Figure S1. Example of two-dimensional statistical total correlation spectroscopy (STOCSY) analysis of 1H-NMR spectrum RAW 264.7 extracts to facilitate the identification of metabolites. (A) 2D STOCSY subplots from 5.8 to 9.4 ppm for the assignments of formate and tyramine; (B) 2D STOCSY subplots from 0.8 to 4.3 ppm for the assignments of dimethylamine and lactate. [file 12906_2020_3115_MOESM1_ESM.tif]

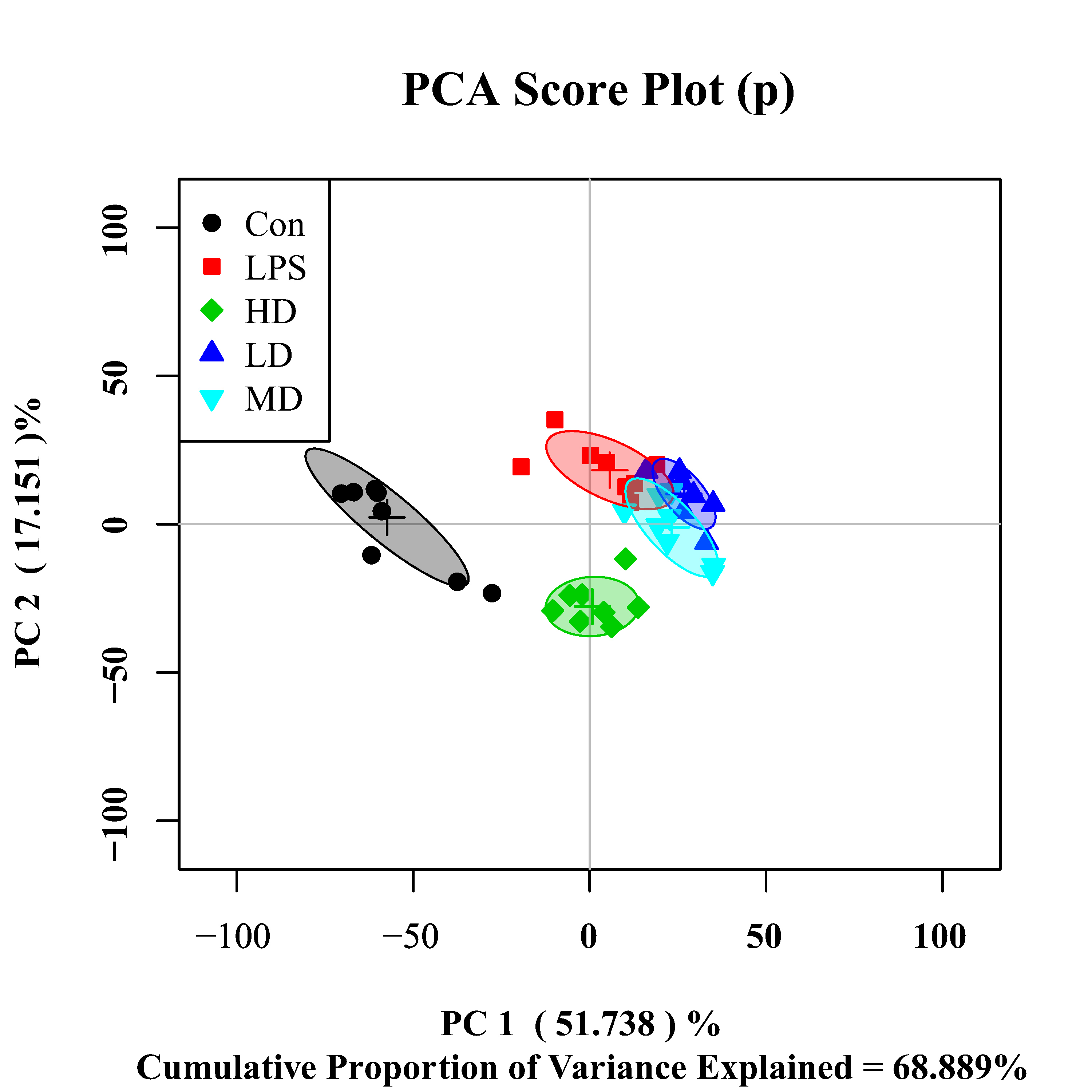

Supplement: Supplementary file 2 — Additional file 2: Figure S2. PCA scores plot for Con, LPS, HD, MD and LD groups. [file 12906_2020_3115_MOESM2_ESM.tif]

yr-65  
2016-3-28 1H-1 CD3OD JAQ-246 (YR-65)

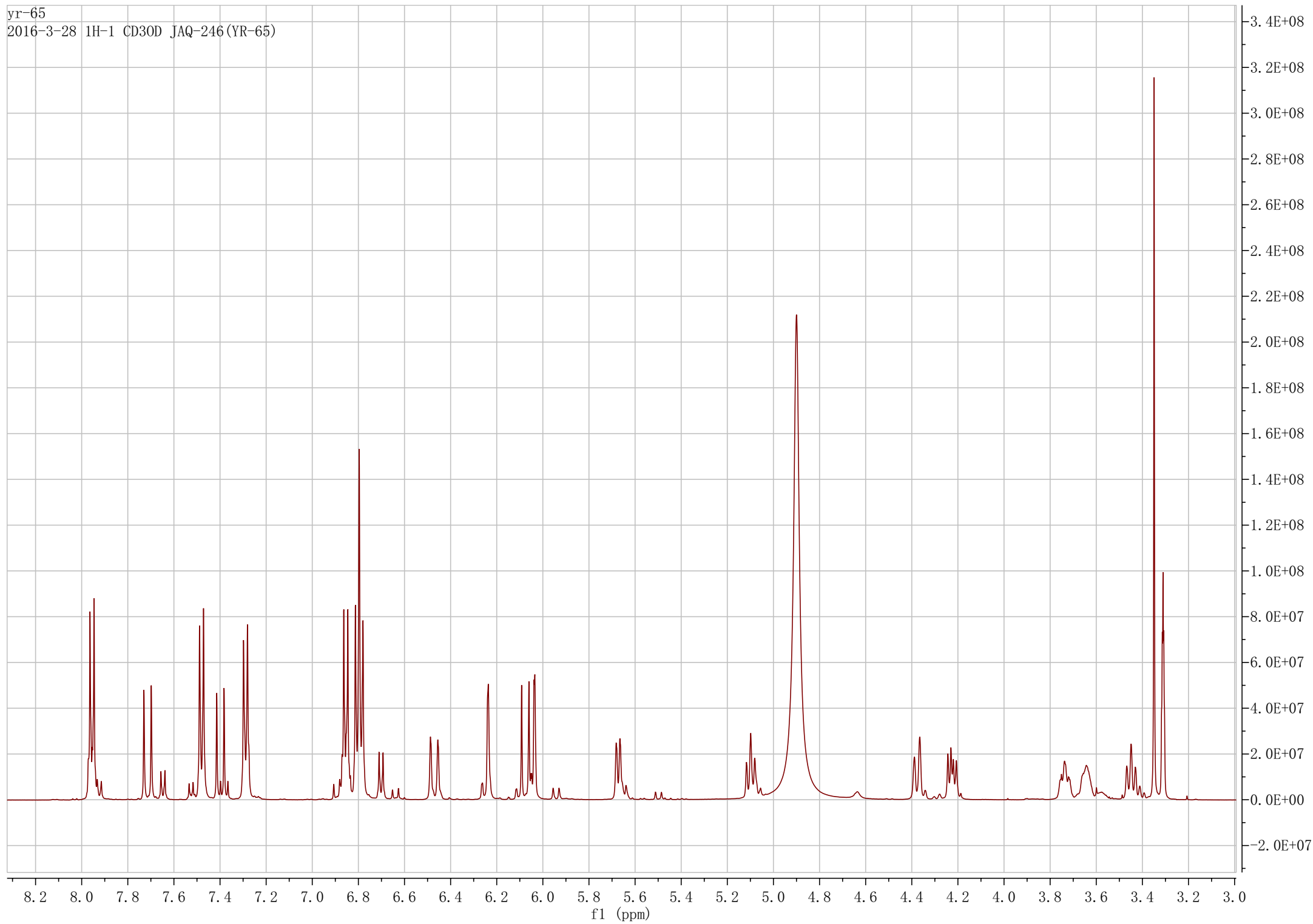

Supplement: Supplementary file 3 — Additional file 3: Figure S3. 1H-NMR spectra of 3-CT. [file 12906_2020_3115_MOESM3_ESM.pdf]

yr-65  
2016-4-3 C13CPD CD30D JAQ-130(YR-65)

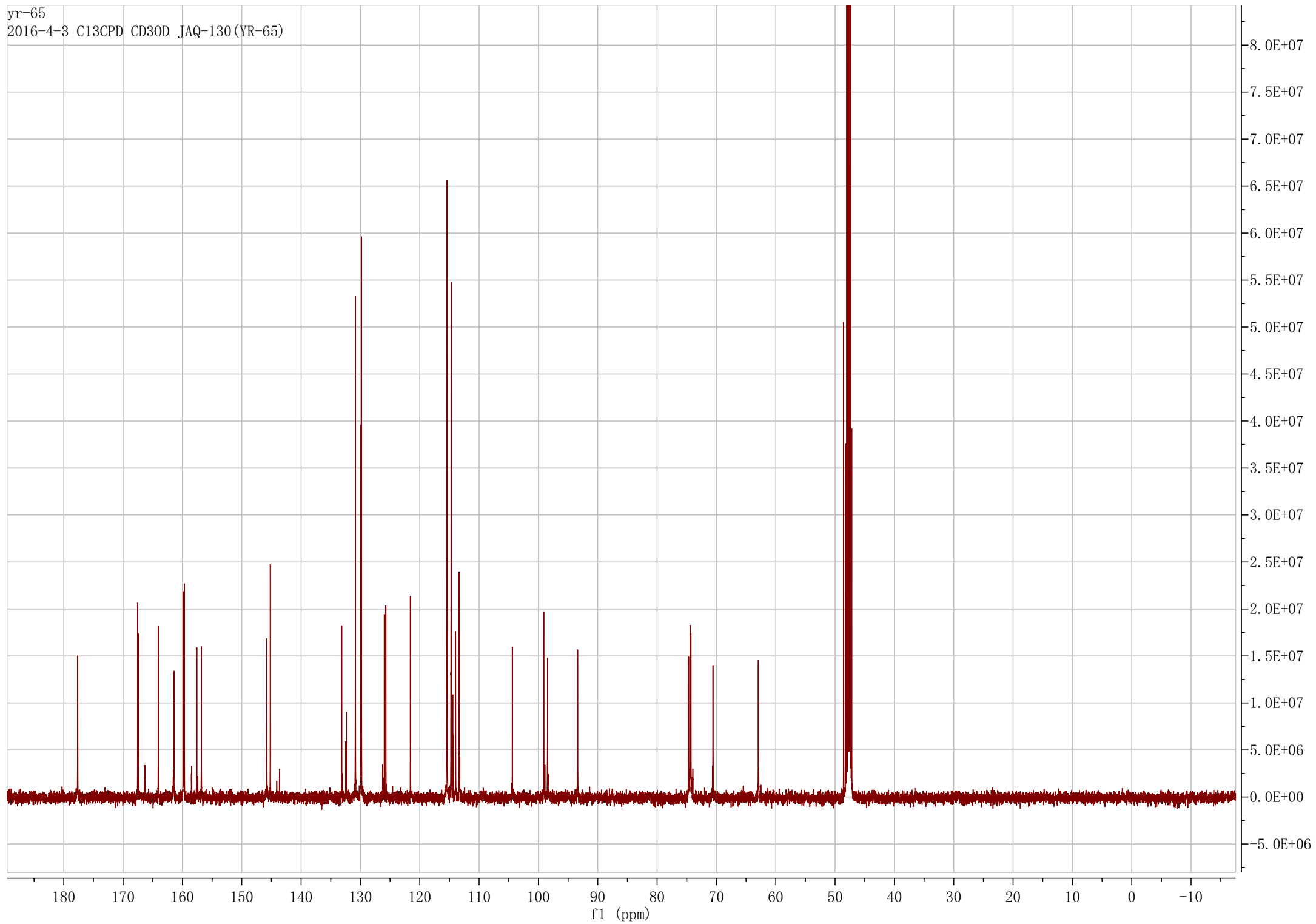

Supplement: Supplementary file 4 — Additional file 4: Figure S4. 13C-NMR spectra of 3-CT. [file 12906_2020_3115_MOESM4_ESM.pdf]
