# Supplementary material for: Anti-inflammatory activity of 3-cinnamoyltribuloside and its metabolomic analysis in LPS-activated RAW 264.7 cells
Source: BMC Complement Med Ther. 2020 Nov 2;20:329. doi: 10.1186/s12906-020-03115-y (PMC7607671; doi:10.1186/s12906-020-03115-y)
Supplement: Supplementary file 5 — Additional file 5: Table S1. The characteristic chemical shifts of metabolites in 1H-NMR spectra. [file 12906_2020_3115_MOESM5_ESM.docx]

Table S1. The characteristic chemical shifts of metabolites in ^1^H-NMR spectra

| No. | Metabolites | Chemical shifts  *δ* ^1^H(ppm) |
| --- | --- | --- |
|  |  |  |
| 1 | 2-Aminobutyrate | 0.97 (q), 1.91 (m),3.71 (t) |
| 2 | Leucine | 0.94 (d), 0.96 (d) |
| 3 | Valine | 0.97 (d), 1.04 (d). |
| 4 | Isoleucine | 0.93 (t), 1.01 (d) |
| 5 | Ethanol | 1.15 (t), 3.64 (q) |
| 6 | Lactate | 1.32 (d),4.10 (q) |
| 7 | Alanine | 1.47 (d), 3.77 (q) |
| 8 | Lysine | 1.72 (m), 3.00 (t) |
| 9 | Acetate | 1.91 (s) |
| 10 | Homoserine | 2.04 (m), 2.14 (m), 3.76 (m), 3.84 (m). |
| 11 | Glutamate | 2.38 (m), 3.75 (m) |
| 12 | Pyroglutamate | 2.03 (m), 2.36 (m), 2.42 (m) |
| 13 | Succinate | 2.41(s) |
| 14 | Glutathione | 2.15 (m), 2.54 (m), 2.96 (m), 3.77 (m) |
| 15 | 5, 6-Dihydrouracil | 2.67 (m), 3.43 (m) |
| 16 | Sarcosine | 2.73 (s),3.61 (s) |
| 17 | Dimethylamine | 2.72 (s) |
| 18 | Creatine | 3.02 (s), 3.92 (s) |
| 19 | Choline | 3.21 (s) |
| 20 | Betaine | 3.25 (s), 3.90 (s) |
| 21 | Methanol | 3.40 (s) |
| 22 | Taurine | 3.25(t), 3.42(t) |
| 23 | Glucose | 3.2-3.9(m),5.2 (d) |
| 24 | Glycine | 3.55(s) |
| 25 | Serine | 3.83 (m), 3.92 (m),3.98 (m) |
| 26 | Cytosine | 5.97 (d) |
| 27 | NAD^+^ | 6.08 (m), 8.42(s) |
| 28 | NADP^+^ | 6.10 (m), 8.20(m) |
| 29 | AMP | 6.14 (d), 8.26 (s), |
| 30 | ATP | 6.15(d), 8.51 (s) |
| 31 | Tyramine | 6.89(d), 7.18 (d) |
| 32 | Histamine | 7.17(m), 7.95 (s), |
| 33 | Phenylalanine | 7.31 (m), 7.42 (m) |
| 34 | Histidine | 7.94 (s) |
| 35 | Formate | 8.4 (s) |

Multiplicity: s, singlet; d, doublet; t, triplet; q, quartet; m, multiplet; br, broad singlet.
